# Exploring Bottom-Up Visual Processing and Visual Hallucinations in Parkinson's Disease With Dementia

**DOI:** 10.3389/fneur.2020.579113

**Published:** 2021-01-28

**Authors:** Nicholas Murphy, Alison Killen, Rajnish Kumar Gupta, Sara Graziadio, Lynn Rochester, Michael Firbank, Mark R. Baker, Charlotte Allan, Daniel Collerton, John-Paul Taylor, Prabitha Urwyler

**Affiliations:** ^1^Faculty of Medical Sciences, Translational and Clinical Research Institute, Newcastle University, Newcastle upon Tyne, United Kingdom; ^2^Menninger Department of Psychiatry and Behavioral Sciences, Baylor College of Medicine, Houston, TX, United States; ^3^ARTORG Center for Biomedical Engineering Research, University of Bern, Bern, Switzerland; ^4^National Institute for Health Research Newcastle In Vitro Diagnostics Co-operative, Newcastle Upon Tyne Hospitals Foundation Trust, Newcastle upon Tyne, United Kingdom; ^5^Gerontechnology and Rehabilitation Group, University of Bern, Bern, Switzerland; ^6^University Neurorehabilitation Unit, Department of Neurology, Inselspital, Bern University Hospital, Bern, Switzerland

**Keywords:** visual processing, Parkinson's disease dementia, visual hallucination, Lewy body, visual evoked potential

## Abstract

Visual hallucinations (VH) are a common symptom of Parkinson's disease with dementia (PDD), affecting up to 65% of cases. Integrative models of their etiology posit that a decline in executive control of the visuo-perceptual system is a primary mechanism of VH generation. The role of bottom-up processing in the manifestation of VH in this condition is still not clear although visual evoked potential (VEP) differences have been associated with VH at an earlier stage of PD. Here we compared the amplitude and latency pattern reversal VEPs in healthy controls (*n* = 21) and PDD patients (*n* = 34) with a range of VH severities. PDD patients showed increased N2 latency relative to controls, but no significant differences in VEP measures were found for patients reporting complex VH (CVH) (*n* = 17) compared to those without VH. Our VEP findings support previous reports of declining visual system physiology in PDD and some evidence of visual system differences between patients with and without VH. However, we did not replicate previous findings of a major relationships between the integrity of the visual pathway and VH.

## Introduction

Visual symptoms are common in Parkinson's disease (PD), and include double vision, dry or painful eyes, poor contrast sensitivity, problems with color vision, and blurring of vision or lowered acuity (1–5). In 45% of PD cases without dementia (6, 7), and up to 65% of cases with dementia (PDD) (8), patients will also experience visual hallucinations (VH). The early presence of VH is a strong predictor of cognitive decline (9), as well as increased mortality and overall reduced quality of life for patients and their carers (10, 11).

Visual dysfunction in PD has been linked to the physical decline of retinal function over the course of disease development due to depletion of retinal dopamine (12), and retinal nerve fiber layer thinning (13). Electrophysiological measures of visual health, such as the visual evoked potential (VEP), and the electroretinogram (ERG), have been widely used to support the diagnosis of PD as indirect measures of health and integrity of early bottom-up visual processing pathways. Measurements of scalp potentials, as well as scotopic alpha and beta waves generated on the retina during foveal stimulation typically demonstrate a slowing of peak activity in PDD patients relative to controls (14–16), acting as indirect support for pathological evidence of a decline in pre-geniculate visual function (12, 13).

Models of VH in Lewy body dementias [including dementia with Lewy bodies (DLB), and PDD] have posited that VH are a product of the inefficient integration of multiple perceptual sub-divisions of the visual system including retinal input (17, 18). Healthy visual perception involves the prediction of sensory inputs expected from the salient features of images (based upon long-term memory of similar images and current context) which are then matched to the actual sensory inputs to minimize any discrepancy between the two. Thus, perception needs to balance predictions and sensory information. Impairments in cognitive control across executive networks in PDD lead to difficulties balancing these processes, thus impairing the accuracy of matching the visual input to expectations. Despite the precise etiology of VH being unclear, variations in the frequency of VH over the course of disease progression suggests that these hallucinations reflect a complex relationship between declining sensory function and dysfunctional predictions (17–22).

In this investigation we sought to characterize the components of early bottom-up processing in PDD patients, using the pattern reversal visual evoked potential, and to relate the response features to the complexity of the VHs experienced. Based on available evidence of physiological decline in PDD we predicted that we would observe a general reduction in the amplitude of the VEP components, as well as an increase in the P1 latency (23). In addition, we expected baseline visual acuity and visual perception, to demonstrate a decline in those with a more severe and frequent complex VH (CVH). This should also extend to an association between VEP P1 and N2 measurements with VH experience, as both of these are thought to be contingent upon attentional and perceptual processes (24, 25), which are, in particular, disrupted by Lewy body pathology (18, 26).

## Methods

### Participants

A total of 21 healthy controls, and 38 Parkinson's disease with dementia (PDD) patients were recruited from the North East of England. Ethical approval was granted by the Newcastle National Health Service (NHS) Health Research Authority (HRA) (REC reference: 13/NE/0252; R&D reference: 6691). The diagnosis of PDD was confirmed by two independent and experienced clinicians (Charlotte Allan, John-Paul Taylor) and met with the standards described in the international PD diagnostic criteria (27). Participants were excluded from the study if baseline assessment revealed the presence of comorbid factors including stroke, non-PD related dementia, and/or visual dysfunction secondary to glaucoma. All procedures related to the study were explained to the participants and written informed consent was obtained prior to participation.

### Clinical Assessments

All participants were assessed on their level of global cognitive function using the Mini Mental State Exam [MMSE, (28); maximum score of 30] and the Cambridge Cognitive Test Battery [CAMCOG total score (29, 30); maximum score of 107]. Motor function was assessed using the total (left and right) score from the Unified Parkinson's disease rating scale section three [UPDRS-III (31); maximum score of 57]. Measurements of fluency and executive functioning were derived from the category fluency test (32) and Trail Making Test A [TMT-A (33)].

The integrity of the participant's visual acuity was assessed using a detailed screening questionnaire, computerized Freiburg acuity testing (34), and the LOGMAR (Logarithm of the Minimum Angle of Resolution) scale of visual acuity. Visuo-perceptual function was assessed using performance on motion sensitivity (35), angle discrimination (35), and performance on the pareidolic imagery test (36).

### Visual Hallucinations

The hallucination subscale (frequency 0 to 4, severity: 0 to 3—not applicable, mild, moderate, marked, and level of distress 0 to 5) of the Neuropsychiatric Inventory (NPIHal) (37) was used for assessing VH occurring in the previous month, with the NPIHal score (frequency × severity of hallucinations) derived as a measure. The NPI-Hall score used in this manuscript is calculated by multiplying the NPI hallucination frequency with NPI hallucination severity. The frequency of hallucination in NPI is coded as 0—not applicable, 1—occasionally (less than once per week), 2—often (about once per week), 3—frequently (several times per week), and 4—very frequently (once or more per day). Severity of hallucination with NPI is scored as 0—not applicable, 1—mild, 2—moderate and 3—marked. For reliability, patients and carers were independently asked about the occurrence of VH in the month before using the North-East Visual Hallucinations Interview (NEVHI) (38). Any discrepancies in the reporting of VH (39) were discussed with both parties and the assessor, with reformulation of NPIHal scores (wherever the patient seemed to lack insight, primacy was given to caregiver opinion).

Participants were classed as active complex visual hallucinators (PD-CVH, *n* = 17) if they had complex VH (CVH) in the month preceding their interview; otherwise, they were classed as non-hallucinators [controls (*n* = 21) and PD-NCVH (*n* = 17)]. Participants with minor VH (e.g., passage or feeling of presence) but no complex VH in the last month were included in the PD-NCVH group. This distinction was made due to the different etiologic basis to CVH even though minor VH typically precede CVH. Patients in this study map onto the same categories used in previously published research from our lab [see (40)]. Additional analysis with a more stringent grouping of PDD-VH (including complex, minor, presence, passage, simple) and PDD-NVH are provided at a later stage in this manuscript.

### EEG

#### Visual Evoked Potential Presentation and Recording

The VEP adhered to the specifications proposed by the International Society for Clinical Electrophysiology of Vision (41) (ISCEV). Participants viewed a black and white checkerboard pattern whilst the checks (visual angle of 0.6°) reversed phase at a rate of 1Hz (switching to the opposite phase every 500 ms), for 200 sweeps, with a brief rest period (3,000 ms) after 100 sweeps. During stimulus presentation a pink dot was placed in the center of the display as a focus point, which the participant was instructed to look at. This was intended to prevent wandering gaze during the check reversal and was presented on top of a gray background during the rest period. The stimulus was generated on a Dell OptiPlex 755 (Microsoft Windows XP) using Matlab v2012a (The MathWorks, 2012), and presented using a Dell U2412M 24-inch LCD monitor (resolution: 1920 × 1,200 pixels refresh rate: 60 Hz). Pattern reversal VEPs were recorded during three separate viewing conditions (both eyes, left eye, right eye), using an ASA-LAB 136 system amplifier and the ASA-LAB recording software (version 4.9.1) in combination with a 128 Ag/AgCl channel Waveguard cap [10-5 system (42), Advanced Neuro Technologies]. The ground electrode was placed on the right clavicle, and Fz was used as the reference electrode. Electrode impedance was kept below 5 kΩ, and no filters were applied during the acquisition of EEG data.

#### Pre-processing

Signal processing and measurement was performed using Matlab v2012a (The MathWorks, 2012), with the EEGLab (43), ERPLab (44), and current source density (45) (CSD) toolboxes. Individual sweeps were split into epochs of 400 ms, a baseline period of 100 ms prior to stimulus presentation, and a post-stimulus period of 300 ms. Epochs were baseline corrected using the mean of the data in the pre-stimulus period and filtered using a 0.1 to 45 Hz bandpass filter. Individual channels with a kurtosis value greater than three standard deviations from the cap-wide mean were removed and recreated after pre-processing using spherical interpolation (43, 46–48). After removing trials containing blinks, muscular activity, and drifting potentials (impedance related artefacts), broad spatial effects of the electric field were attenuated by applying a Laplacian transform (45). This approach was applied to reduce the likelihood of false positives in spatially distant locations when defining the occipital region of interest (ROI).

#### Measurement

To account for individual variance in the timing of synaptic communication the VEP components were measured within windows defined by the global field power (GFP) for each individual. The VEP components elicited three GFP maxima following stimulus presentation, each of which was used as the center point for the corresponding component window (GFP maxima ±10 ms). The occipital ROI was defined by measuring the amplitudes of the P1 component for the grand average of the control data set and using the 20 electrodes with the greatest amplitude as the limit for the ROI. Individual subject measurements of peak latency and mean amplitude were taken from the average VEP waveform within the occipital ROI. To account for potential inter-ocular latency differences, we estimated the difference between P1 peak latency measurements for the left and right eyes. Three separate recordings were taken from each participant: viewing with both eyes; viewing with left eye; viewing with right eye. The three recordings were used to help screen for inter-ocular variations that could have indicated undocumented eye disease. The analyses were performed using the both eyes recording.

#### Statistical Analysis

All statistical tests were performed using the Statistical Package for the Social Sciences (SPSS, version 22). For all comparisons the data were inspected for violations of normality using the Shapiro-Wilk test due to it being more sensitive to small sample sizes. Demographic and baseline factors were compared using independent samples *t*-tests. We compared the measurements of amplitude and latency separately for each component using univariate analysis of variance controlling for age and interocular latency difference between the left and right eyes. As further validation of the findings, we expanded our model to include UPDRS, CAMCOG, and L-Dopa dose as covariates. Effect sizes were estimated using the partial eta squared measure (η^2^). To explore the relationship between the variance within our physiological measurements and VH experience in the hallucinating PDD group, we performed parametric correlations between each VEP measurement and NPI hallucination score (NPIHal) and NPI hallucination severity. To help identify any variance in our measurements accounted for by clinical and/or demographic factors we performed additional parametric correlations between the VEP measurements and each value. To investigate the contribution of the minor VH, all the above-mentioned tests were explored with regrouping PDD-NVH vs. PDD-VH. Significance for all tests was determined using an alpha criterion of *p* < 0.05, and Bonferroni corrected for multiple comparisons (corrected alpha criterion of *p* < 0.016). Where appropriate un-corrected correlations are reported to highlight trends within individual results.

## Results

### Demographics and Clinical Scores

Demographic results are summarized in Table 1. All groups were matched for age and there were no significant differences in duration of PD (*p* = 0.09) or levodopa dose (*p* = 0.27) between the PDD-CVH and PDD-NCVH groups. PDD patients displayed a significant reduction in global cognitive function, UPDRS motor score relative to controls, with the PDD-CVH group global cognitive function and motor function were significantly worse when compared to the PDD-NCVH group. The NPI recorded the severity of hallucinations as mild in 29.4% (5 of 17) PDD-NCVH which were phenomenologically classified as Illusions (3 of 5, 75%), presence (4 of 5, 66.7%), shadow (3 of 5, 75%), and simple (3 of 5, 75%) by the NEVHI. Further stringent grouping into PDD_NVH dropped the reporting of mild hallucinations to 10%.

**Table 1:** Participant demographics and clinical scores.

**Measurement**	**Controls**	**PDD NCVH**	**PDD CVH**	***Post-hoc***	***Statistics***
	**(*n =* 21)**	**(*n =* 17)**	**(*n =* 17)**		***Test Val, p, Effect Size***
Age (years)	74.95 ± 5.16	72.94 ± 5.19	73.88 ± 5.36		0.7, 0.501, 0.026
MMSE score	29.1 ± 1.81	24.59 ± 5	22.76 ± 4.99	HC>NCVH, HC>CVH	12.307, <0.001*, 0.321
CAMCOG total score	95.14 ± 6.79	80.94 ± 15.53	73.18 ± 15.91	HC>NCVH, HC>CVH	14.003, <0.001*, 0.350
CAMCOG Executive score	22.29 ± 3.16	14.53 ± 3.94	12.65 ± 4.08	HC>NCVH, HC>CVH	36.84, <0.001*,0.59
Trial Making Test A (sec)	34.1 ± 10.81	71.94 ± 41.98	116.64 ± 100.84	HC < CVH	8.221, 0.001*, 0.260
Categorial Fluency(animals/min)	18.24 ± 5.09	12.65 ± 3.64	10.44 ± 5.73	HC>NCVH, HC>CVH	12.730, <0.001*, 0.333
UPDRS III score	2.10 ± 2.47	38.65 ± 21.93	57.88 ± 20.48	HC < NCVH, HC < CVH, CVH>NCVH	55.147, <0.001*, 0.680
Acuity (decimal)	0.57 ± 0.51	0.29 ± 0.47	0.19 ± 0.4	HC>CVH	3.403, 0.041*, 0.118
Acuity (logmar)	0.31 ± 0.26	0.65 ± 0.33	0.58 ± 0.27	HC>NCVH, HC>CVH	7.823, 0.001*, 0.235
Minimum Angle Perception (degrees)	8.64 ± 3.25	28.42 ± 23.51	32.68 ± 30.07	HC < NCVH, HC < CVH	7.058, 0.002*, 0.214
Motion Perception	−2.72 ± 0.72	1.80 ± 3.15	2.68 ± 2.88	HC < NCVH, HC < CVH	26.746, <0.001*, 0.522
Number of Pareidolia	1.0 ± 1.46	3.18 ± 4.54	6.82 ± 5.58	HC < CVH, CVH>NCVH	8.188, 0.001*, 0.254
Interocular difference (ms)	4.82 ± 3.9	4.35 ± 4.4	9.44 ± 7.64	HC < CVH, CVH>NCVH	4.536, 0.015*, 0.151
Levodopa Dose (24 h, mg)^#^		569.12 ± 303.05	710.59 ± 363.10		−1.23, 0.27, 334.43
PD Duration (years)^#^		7.12 ± 4.57	10.82 ± 7.46		−1.75, 0.09, 6.19
NPIHal (frequency × severity)^#^ score		0.29 ± 0.59	3.13 ± 2.13	CVH>NCVH	−5.29, <0.001*, 1.54
NPI hallucination severity (*n*, none/mild/moderate)		12/5/0	0/14/3		${\text{χ}}_{\left(2\right)}^{2}$ = 19.263, <0.001*
NPI hallucination frequency (n, none/occasional/often/frequent/daily)		12/4/1/0/0	0/3/4/5/5		${\text{χ}}_{\left(4\right)}^{2}$ = 23.943, <0.001*

Data are mean ± standard deviation. Statistical tests: Univariate analysis of variance (ANOVA) with partial eta squared effect size,

# independent samples t-tests and Cohen's D effect sizes, p–value 2 sided <0.05 significant (*); PD, Parkinson's disease; PDD, Parkinson's disease dementia; MMSE, Mini-Mental State Examination; CAMCOG, Cambridge Cognitive Assessment; UPDRS, Unified Parkinson's Disease Rating Scale; HC, Healthy Controls; CVH, Complex Visual Hallucination; NCVH, No Complex Visual Hallucination. Bold values indicate statistically significant values.

### Visual Integrity and Visual Perceptual Scores

Cataracts were reported by 20% (Controls 6.9%, PDD-NCVH 3.4%, PDD-CVH 10.3%) of the recruited participants while 19% of the participants had cataracts removed (Controls 5.2%, PDD-NCVH 6.9%, PDD-CVH 6.9%). Other ophthalmological history reported include glaucoma by two participants (PDD-VH 1.7%, HC 1.7%), macular degeneration by one participant (HC 1.7%), and laser treatment (HC 1.7%).

Visual acuity and perceptual scores are summarized in Table 1. There was a pattern of overall decline in visual integrity within the PDD patients relative to the control group, characterized by a significant reduction in LOGMAR (*p* = 0.001) measurements of visual acuity. As expected, PDD-CVH patients showed a characteristic significant increase in the number of false perceptions (*p* = 0.047) reported during the pareidolia task compared to PDD-NCVH patients. Interocular latency differences were significantly higher in PDD-CVH compared to the PDD-NCVH (*p* = 0.028) group.

### Visual Evoked Potential

**Figure 1:**
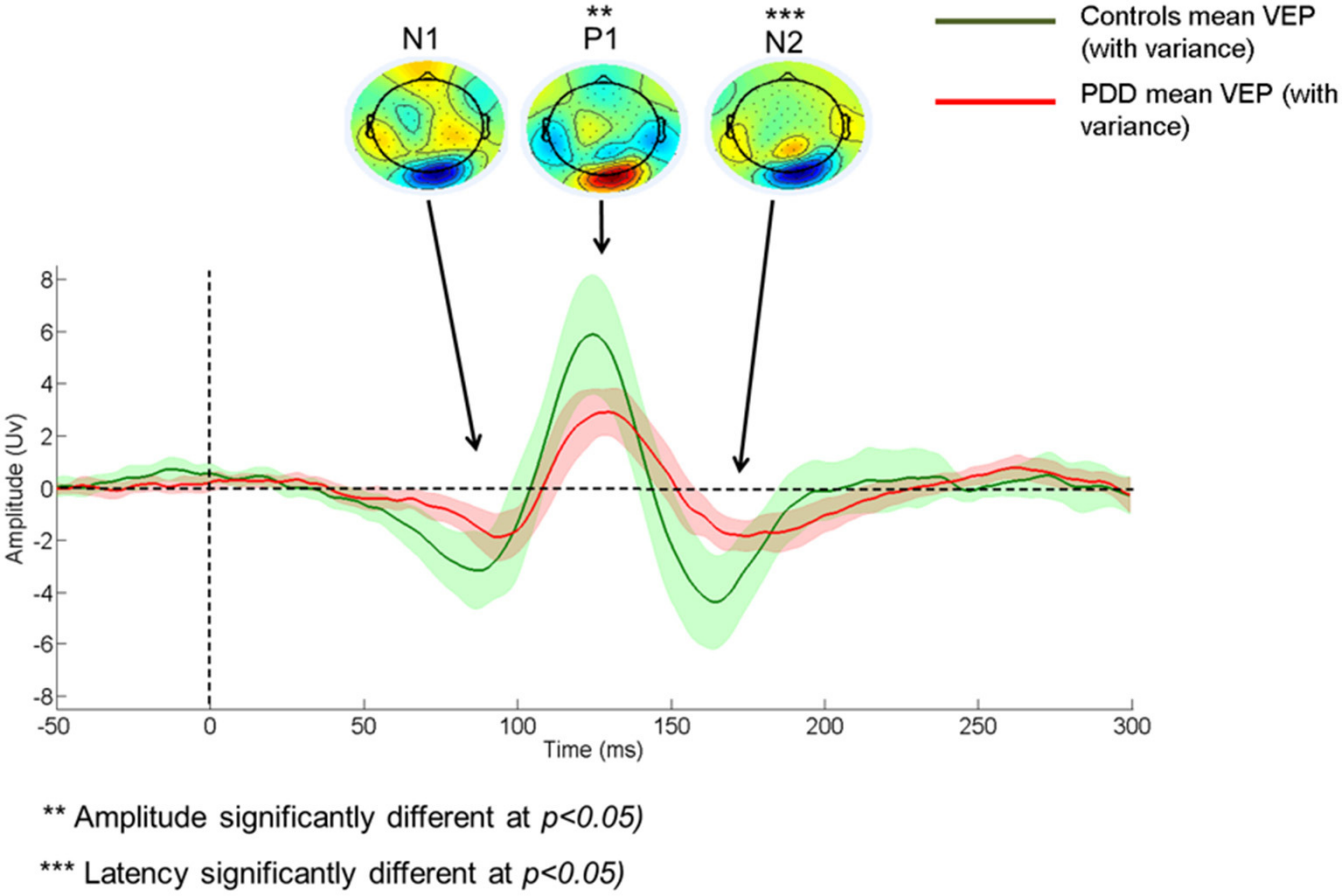
Comparison of Control and Patient VEP waveform. PDD, Parkinson's disease dementia; VEP, Visual evoked potential; VEP components - N1, P1, and N2.

**Figure 2:**
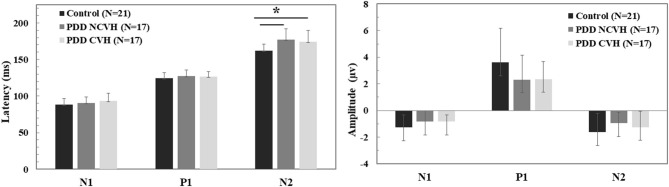
Comparison of the visual evoked potential component (N1, P1, and N2) amplitude and latency. Statistical tests: Univariate analysis of variance (ANOVA), df = 52, *p*–value 2 sided <0.05 significant; PDD, Parkinson's disease dementia; HC, Healthy Controls; CVH, Complex Visual Hallucination; NCVH, No Complex Visual Hallucination; *Post-hoc = CVH>HC, NCVH>HC.

Amplitude tended to be smaller, and latency later in PDD vs controls (*Amplitude* N1: −0.844 ± 0.67 vs. −1.27 ± 0.93, P1: 2.34 ± 1.57 vs. 3.61 ± 2.55, N2: −1.12 ± 1.02 vs. −1.64 ± 1.44; *Latency* N1: 91.79 ± 9.60 vs. 88.28 ± 8.62; P1: 127.21 ± 6.96 vs. 124.50 ± 7.31, N2: 175.54 ± 14.84 vs. 162.26 ± 8.98), although this was only significant for P1 and N2 components. A visual representation of the VEP is presented in Figure 1. There were no significant differences between PDD-CVH vs. PDD-NCVH. (see Figure 2) for the VEP latencies and amplitudes (N1: *p* = 0.210; P1: *p* = 0.120), except N2 latency (Controls: 162.27 ± 8.98, PDD-NCVH: 176.93 ± 14.69, PDD-CVH:174.15 ± 15.30, *F* = 7.95, *p* = 0.001, η^2^ = 0.241). A *post hoc* Bonferroni comparison indicated that N2 latency in controls was significantly less than PDD-NCVH group (*p* = 0.02) and PDD-CVH (*p* = 0.018), but the N2 latency did not differ between the CVH and NCVH group while controlling for age and interocular latency differences. Further validation result indicates no significant effect between PDD_NCVH and PDD_CVH on latency and amplitude of N1, P1, and N2 components even after controlling for UPDRS, CAMCOG, and L-Dopa dose along with age and interocular latency.

Additional analysis with a stringent regrouping of PDD-NVH and PDD-VH show similar results (Table 2) as in Figures 1, 2. *Post hoc* Bonferroni comparison indicated that N2 latency in controls was significantly less than PDD-NVH group (*p* = 0.029) and PDD-VH (*p* = 0.001), but the N2 latency did not differ between the VH and NVH group. The regrouping of minor VH into the PDD-VH group shows a trend toward significant difference for the N1 (*p* = 0.072) and P1 (*p* = 0.099) amplitude.

**Table 2:** Comparison of the visual evoked potential components across groups [grouping with any visual hallucination (VH)].

**Component**		**Controls (*n =* 21)**	**PDD-NVH (*n =* 11)**	**PDD-VH (*n =* 23)**	**Statistics F, *p*-value, **η^2^****
N1	Amplitude (μv)	−1.27 ± 0.93	−1.14 ± 0.85	−0.70 ± 0.53	2.78, 0.072, 0.100
	Latency (ms)	88.28 ± 8.62	90.61 ± 10.41	92.35 ± 9.38	0.998, 0.376, 0.038
P1	Amplitude (μv)	3.61 ± 2.55	2.82 ± 1.93	2.11 ± 1.35	2.425, 0.099, 0.088
	Latency (ms)	124.50 ± 7.32	128.76 ± 6.56	126.47 ± 7.17	1.978, 0.149, 0.073
N2	Amplitude (μv)	−1.64 ± 1.44	−1.17 ± 0.88	−1.09 ± 1.10	0.843, 0.436, 0.033
	Latency (ms)	162.27 ± 8.97	174.33 ± 10.84	176.12 ± 16.61	7.787, 0.001^*^, 0.238

Data are mean ± standard deviation. Statistical tests: Univariate analysis of variance (ANOVA), df = 52, p–value 2 sided <0.05 significant; PDD, Parkinson's disease dementia; HC, Healthy Controls; VH, Visual Hallucination (includes simple, passage, presence, and complex); NVH, No Visual Hallucination;

* *Post-hoc = VH>HC, NVH>HC. Bold values indicate statistically significant values*.

### Clinical Correlations

Clinical correlation of VEP components for latency and amplitude are shown in Tables 3, 4, respectively. Hallucination experience as quantified using the NPIHal subscale score (frequency × severity) was not significantly related to the measurements of any of the VEP components. However, VH severity assessed using the NPI hallucination severity showed significant negative correlation with P1 latency in PDD-NCVH patients (*r* = −0.492, *p* = 0.045) and P1 amplitude in PDD-CVH patients (*r* = −0.555, *p* = 0.021).

**Table 3:** Correlation of visual evoked potential components (N1, P1, and N2 Latency) with clinical variables.

	**PDD (*****N*** **=** **34)**			**PDD-NCVH (*****N*** **=** **17)**			**PDD-CVH (*****N*** **=** **17)**		
	**N1**	**P1**	**N2**	**N1**	**P1**	**N2**	**N1**	**P1**	**N2**
Age	0.144	0.300	0.168	0.190	0.161	0.040	0.084	0.482^*^	0.308
CAMCOG total score	−0.181	−0.150	−0.210	−0.286	−0.179	−0.200	−0.034	−0.161	−0.282
CAMCOG Executive score	−0.081	−0.235	−0.298	−0.171	−0.334	−0.342	0.057	−0.166	−0.322
Trail making Test A	−0.608	−0.014	−0.027	0.290	0.555^*^	0.348	−0.252	**–**0.259	−0.137
Animal Categorial Fluency	−0.394^*^	–0.396^*^	−0.565**	−0.253	−0.328	0.619**	−0.447	−0.541^*^	−0.652**
UPDRS III score	−0.034	0.115	0.013	0.087	0.124	0.081	−0.299	0.190	0.036
Acuity (decimal)	0.084	0.009	−0.073	0.119	0.078	0.092	0.051	−0.166	−0.248
Acuity (logmar)	−0.058	−0.040	0.100	−0.118	−0.104	−0.071	0.040	0.035	0.286
Minimum Angle Perception	−0.407^*^	−0.206	−0.487**	−0.081	−0.267	−0.444	−0.648^*^	−0.158	−0.516
Motion Perception	−0.063	−0.036	−0.148	0.163	0.113	0.219	−0.216	−0.212	−0.448
Number of Pareidolia	0.014	0.086	−0.216	0.149	0.063	−0.347	−0.179	0.166	−0.078
Interocular difference	0.197	−0.169	−0.074	−0.211	−0.043	0.030	0.321	−0.205	0.035
Levodopa Dose	−0.096	−0.064	−0.051	−0.022	0.274	0.080	−0.215	−0.389	−0.122
PD Duration	−0.187	0.098	0.049	−0.049	0.062	−0.212	−0.354	0.174	0.253
NPI hallucination score	−0.031	−0.301	−0.217	−0.127	−0.254	0.333	−0.196	−0.454	−0.358
NPI hallucination severity	0.063	−0.256	−0,083	−0.234	−0.492^*^	0.025	0.063	−0.057	−0.074

Data are Pearson correlation, p–value 2 sided significant at the **0.01 level,

* *0.05 level; PDD, Parkinson's disease dementia; CVH, Complex Visual Hallucination; NCVH, No Complex Visual Hallucination. Bold values indicate statistically significant values*.

**Table 4:** Correlation of visual evoked potential components (N1, P1 and N2 Amplitude) with clinical variables.

	**PDD (*****N*** **=** **34)**			**PDD-NCVH (*****N*** **=** **17)**			**PDD-CVH (*****N*** **=** **17)**		
	**N1**	**P1**	**N2**	**N1**	**P1**	**N2**	**N1**	**P1**	**N2**
Age	−0.145	0.232	−0.271	−0.115	0.262	−0.275	−0.206	0.198	−0.257
CAMCOG total score	−0.152	0.137	−0.038	−0.321	0.277	−0.229	0.093	−0.027	0.027
CAMCOG Executive score	−0.370^*^	0.286	−0.089	−0.564^*^	0.458	−0.358	−0.122	0.096	0.026
Trail making Test A	0.117	−0.073	0.020	0.332	−0.261	0.282	−0.041	0.049	−0.021
Animal Categorial Fluency	0.004	−0.042	0.172	−0.203	0.208	−0.165	0.223	−0.263	0.283
UPDRS III score	0.300	−0.189	−0.070	0.254	−0.151	0.088	0.473	−0.316	−0.086
Acuity (decimal)	−0.161	0.168	−0.068	−0.030	0.103	−0.228	−0.235	0.136	0.165
Acuity (logmar)	0.094	−0.114	0.069	−0.019	−0.049	0.168	0.335	−0.230	−0.047
Minimum Angle Perception	0.185	−0.066	−0.163	0.104	0.033	−0.015	0.443	−0.281	−0.171
Motion Perception	0.003	−0.061	0.100	0.003	−0.106	0.257	0.000	−0.046	0.087
Number of Pareidolia	0.227	−0.121	−0.131	0.063	0.068	−0.101	0.504^*^	−0.370	−0.081
Interocular difference	0.024	−0.101	0.124	−0.038	0.042	−0.105	0.109	−0.270	0.336
Levodopa Dose	0.085	0.013	−0.042	0.131	−0.084	0.225	0.034	0.117	−0.151
PD Duration	0.086	0.098	−0.403^*^	0.093	0.031	−0.022	0.104	0.168	−0.536^*^
NPI hallucination score	0.110	−0.124	0.008	0.216	−0.256	0.228	0.226	−0.284	0.171
NPI hallucination severity	0.185	−0.177	0.027	0.164	−0.101	0.054	0.458	−0.555^*^	0.322

Data are Pearson correlation, p–value 2 sided significant at the **0.01 level,

* *0.05 level; PDD, Parkinson's disease dementia; CVH, Complex Visual Hallucination; NCVH, No Complex Visual Hallucination. Bold values indicate statistically significant values*.

Minimum angle of perception demonstrated significant negative correlation with N1 (*r* = −0.407, *p* = 0.028) and N2 (*r* = −0.487, *p* = 0.007) latency in PDD patients and was also significant for N1 latency in PDD-CVH patients (*r* = −0.648, *p* = 0.012). False perception in Pareidolia tasks demonstrated significant positive correlation with N1 amplitude in PDD-CVH (*r* = 0.504, *p* = 0.039) patients. Interocular latency showed no significant correlations with NPI hallucination severity (*r* = −0.280, *p* = 0.220), and NPI hallucination frequency (*r* = −0.119, *p* = 0.606).

Executive scores such as CAMCOG Executive demonstrated significant correlations with N1 amplitude in PDD patients **(***r* = −0.370, *p* = 0.031) and PDD-NCVH patients **(***r* = −0.564, *p* = 0.018), TMT-A with P1 latency in PDD-NCVH (*r* = 0.555, *p* = 0.026) patients; and categorial fluency scores with all VEP latency components in PDD patients (N1: *r* = −0.394, *p* = 0.023, P1: *r* = −0.396, *p* = 0.023, N2: *r* = −0.565, *p* = 0.001) followed with latency significance in PDD-CVH patients (P1: *r* = −0.541, *p* = 0.030, N2: *r* = −0.652, *p* = 0.006) and PDD-NCVH patients **(**N2: *r* = −0.619, *p* = 0.008).

In PDD-CVH patients, significant negative correlation was found for N2 amplitude with the duration of PD (*r* = −0.536, *p* = 0.026) and P1 latency with age (*r* = 0.482, *p* = 0.050). Correlation analysis with regrouping into PDD-NVH and PDD-VH as shown in Table 5 do not change the direction of our results.

**Table 5:** Correlation of visual evoked potential components (N1, P1 and N2) with clinical variables (patients with VH and NVH).

	**Latency**						**Amplitude**					
	**PDD-NVH (*****N*** **=** **11)**			**PDD-VH (*****N*** **=** **23)**			**PDD-NVH (*****N*** **=** **11)**			**PDD-VH (*****N*** **=** **23)**		
	**N1**	**P1**	**N2**	**N1**	**P1**	**N2**	**N1**	**P1**	**N2**	**N1**	**P1**	**N2**
Age	0.190	0.062	0.081	0.118	0.424^*^	0.208	−0.165	0.385	−0.470	−0.141	0.123	−0.193
CAMCOG total score	−0.529	−0.394	0.154	−0.039	−0.167	−0.276	−0.042	−0.085	0.240	−0.070	0.149	−0.093
CAMCOG Executive score	−0.060	−0.050	0.373	−0.065	−0.369	−0.477^*^	−0.560	0.346	−0.132	−0.188	0.203	−0.066
Trail making Test A	0.246	0.370	−0.095	−0.166	−0.023	−0.023	0.101	−0.078	−0.014	0.016	−0.001	0.012
Animal Categorial Fluency	−0.340	−0.343	−0.136	−0.436^*^	−0.501^*^	−0.643**	0.173	−0.281	0.417	0.080	−0.063	0.154
UPDRS III score	0.149	0.398	0.118	−0.172	0.112	−0.043	0.194	−0.073	−0.054	0.232	−0.154	−0.105
Acuity (decimal)	0.307	0.058	0.248	−0.040	0.005	−0.179	−0.101	0.306	−0.489	−0.273	0.116	0.083
Acuity (logmar)	−0.262	−0.089	−0.202	0.079	−0.046	0.225	0.044	−0.212	0.393	0.225	−0.093	−0.062
Minimum Angle Perception	−0.171	0.245	0.187	−0.500^*^	−0.230	−0.597**	0.740^*^	−0.804**	0.779^*^	0.087	0.083	−0.282
Motion Perception	0.039	0.293	0.226	−0.067	−0.096	−0.279	−0.117	−0.170	0.384	−0.100	0.106	−0.013
Number of Pareidolia	0.161	0.115	−0.195	−0.073	0.107	−0.237	0.295	−0.158	0.084	0.142	−0.061	−0.215
Interocular latency difference	−0.174	−0.194	0.405	0.315	−0.140	−0.179	0.179	−0.110	0.024	−0.121	−0.064	0.143
Levodopa Dose	−0.201	−0.329	−0.569	−0.075	0.048	0.057	−0.031	0.061	0.051	0.093	0.036	−0.075
PD Duration	0.096	0.031	−0.134	−0.330	0.182	0.066	−0.002	0.081	−0.065	0.002	0.218	−0.516^*^
NPI hallucination score	−0.450	−0.042	0.054	−0.061	−0.279	−0.314	−0.106	0.107	−0.085	−0.087	−0.040	−0.006
NPI hallucination severity	−0.450	−0.042	0.054	0.144	−0.259	−0.229	−0.106	0.107	−0.085	−0.052	−0.093	0.015

Data are Pearson correlation, p–value 2 sided significant at the **0.01 level,

* *0.05 level; PDD, Parkinson's disease dementia; VH, Visual Hallucination; NVH, No Visual Hallucination. Bold values indicate statistically significant values*.

## Discussion

In healthy participants, the VEP reflects a combination of many pre-striate and cortical processes. It is noted that a decline in visual pathway integrity following structural changes to the retina can affect the latency and amplitude (16, 49–51). In earlier studies the VEP has consistently been shown to be affected by PD neuropathology, indicating substantial decline in the quality of bottom-up visual processing (3, 14, 16). Following the hypothesis that disrupted bottom-up processing of visual input is associated with the generation of VH in PDD we investigated whether the VEP could be used as a marker of hallucination symptomology.

In accordance with previous research (3, 27, 52–54) the PDD patients demonstrated a reduction in visual acuity, impaired visual perception, impoverished motor ability, and compromised global cognition. Analysis of the pattern reversal VEP data revealed a significant increase in the PDD N2 latency relative to controls, especially in PDD-CVH, and non-significant reduction in the PDD P1 amplitude.

P1 and N2 (N140) are both linked to physical properties of the stimulus such as luminance, brightness, position on the retina, and associated attentional demands (55–60). Further, the N2 (N140) has been reported to be associated with increased disease severity (61). This is reflected in our significant correlation of N2 amplitude with the duration of PD in PDD with complex VH.

Significant differences in the interocular latency between the CVH and NCVH suggest a difference in low level visual processing between the two groups. Intact low-level visual processing is required to differentiate the different types of inputs projected onto the retina. These interocular differences might be explained by changes within the eye, such as retinal thinning, which is well documented in PD patients (and more prominent in those that experience VH) (62). It is known that processing delays between the two eyes can result in an illusory percept (63). Our results thus support greater asymmetry of ocular processing to be involved in CVH. However, no significant correlations were found with the NPI hallucination severity. In future, this is a feature worth studying in more detail, including computational and animal models of PDD macular degeneration and VH.

In patients with PDD there are often abnormalities associated with the structure and function of the retina, including changes in morphology and dopaminergic signaling (3), which have previously been linked to reduced conduction velocity in early visual processing (16, 64–69). Source localization of these components places the generating sources deep within the secondary visual cortex (70, 71); although their cognitive associations suggest that their activity is governed as part of a higher order visual processing network.

However, our findings are at odds with our hypothesis based on previous work of increased P1 latency (23) in PD with VH. This discrepancy may have arisen because the groups in Matsui et al. were matched on cognitive scores, whereas our two PDD groups did differ on cognitive measures, with more impairment in the PDD-CVH group including perceptual impairment. This is supported by NPI hallucination severity correlating with P1 latency in PDD-NCVH patients and P1 amplitude in PDD-CVH patients (*r* = −0.555, *p* = 0.021). The inclusion of minor VH into the PDD-NCVH group potentially explains the correlation with P1 amplitude. Moreover, attentional and perceptual measures (angle detection and pareidolia task) were related to N1 latency and N1 amplitude in patients with complex VH. These reinforce the argument that whilst bottom up dysfunction places individual at risk of VH, it is disruption of these top down processes which are needed for the manifestation of hallucinations.

Given the extent of association between the VEP components and major clinical measurements in our study, it is clear that there is a deliberate pattern of communication that occurs between the primary visual cortex and both its bottom-up and top-down projections. However, our experimental design is limited in the scope to which we can draw conclusions on the nature of pathological change within the executive system and the link between attention and passive perception of the VEP stimulus.

In the context of a mechanism for VH, our sample results suggest that bottom-up processing is not differentially affected between hallucinators and non-hallucinators. This is not unexpected as it follows that in an integrative model of VH we would expect VH content to stem from the interaction of impaired bottom-up processing with dysfunctional top-down control of perception. In our data, complex VH were associated with greater decline in CAMCOG and UPDRS scores, as well as increased numbers of pareidolia relative to patients without complex VH. The divergence in the cognitive and perceptual profile of the groups supports a deteriorated capacity for effective top-down control, which in this model would be a pre-requisite factor for the generation of complex VH. However, these measures were not significantly correlated with the amplitude or latency of the VEP component measurements suggesting that conduction velocity and basic processing of visual feature information is unimpeded by the integrity of detailed perceptual processing. Our findings suggest that the integrity of visual processing has a complex interaction with the higher perceptual function. To build a stronger mechanistic model of this interaction, future work should study the VEP at its cortical sources, whilst also incorpating structural and functional magnetic resonance imaging data.

Within the integrative model of complex VH in Lewy body dementias the importance of bottom-up processing is thought to be its influence on the generation of proto-objects (17, 18). The frequency and phenomenology of the VH would then depend on the interaction between the executive system and the perceptual processing centers. Therefore, declining visual health and perceptual quality might simply place the individual in an at-risk state for VH development (40) rather than directly impact their generation. However, from the results presented we can gleam that Lewy body disorders play a substantial role in the generation of visual hallucinations. This is demonstrated by relationships with both cognition when vision is controlled for, and vice-versa. The lack of a substantive direction for the relationship between VH complexity and bottom-up and top-down factors suggests that the risk for VH development is, therefore, more complicated than we had hypothesized. Future research will be required to provide a detailed assessment of the relative contributions of these factors and will require a study design that deliberately varies the cognitive and visual profiles across samples.

### Limitations

There are several limitations. Firstly, the sample size within this study was relatively small, which does not allow for strong conclusions. In future, we will use the data to design larger confirmatory studies. Secondly, we used only a single subjective measure for VH severity. The NPI items are typically collected from the carers of the patient, and do not ask questions about the content of the hallucination. It thus remains possible that there could be a relationship between visual health, bottom-up processing, and VH content that could be accessed by quantifying a scale such as the North East Visual Hallucination Interview (NEVHI) (38). Furthermore, the range of VH severity scores in our groups was limited making correlative analyses more difficult.

## Conclusion

In summary, PDD patients demonstrated a diminished profile for visual information processing by way of lowered acuity and reduced visual integrity. This was partially reflected in the outcome of the VEP components, although the broad lack of significant differences between PDD-CVH, PDD-NCVH, and healthy controls implies that bottom-up visual information processing remains reasonably intact. Our findings suggest that while bottom-up processing is not grossly affected by the stage of PDD there is a complex interaction between cognitive, visual, and physiological aspects of visual processing in the generation of VH. To advance our understanding in this field, our findings also support a separation between bottom-up information processing and the mechanism of complex VH generation, and instead imply that the reduced visual integrity might act to place the individual in an at risk state for the development of hallucinations in patients with a deteriorated cognitive profile. Thus, a main contribution to this field of work, lies in redirecting research from low level visual dysfunction to higher level processes. Future work should focus on a multimodal approach to understanding the interactions between top-down and bottom-up perceptual circuitry and how this is impacted by PDD neuropathology.

## Data Availability Statement

The raw data supporting the conclusions of this article will be made available by the authors, without undue reservation.

## Ethics Statement

The studies involving human participants were reviewed and approved by Newcastle National Health Service (NHS) Health Research Authority (HRA). The patients/participants provided their written informed consent to participate in this study.

## Author Contributions

NM, SG, LR, MRB, CA, DC, and J-PT contributed to the conception and organization of the research. NM and AK participated in the execution and data collection. NM, PU, and MF designed and implemented the data analysis and interpretation. NM and PU wrote the first draft of the manuscript. RG implemented the data analysis and interpretation for the revision. All authors approved the final version of this manuscript.

## Conflict of Interest

The authors declare that the research was conducted in the absence of any commercial or financial relationships that could be construed as a potential conflict of interest. The reviewer DF declared past co-authorship and research collaboration with several of the authors DC, J-PT, PU, MF to the handling Editor.
